# A contribution to the knowledge of cavernicolous ground beetles from Sichuan Province, southwestern China (Coleoptera, Carabidae, Trechini, Platynini)

**DOI:** 10.3897/zookeys.1008.61040

**Published:** 2020-12-31

**Authors:** Mingyi Tian, Li He

**Affiliations:** 1 Department of Entomology, College of Plant Protection, South China Agricultural University, 483 Wushan Road, Guangzhou, 510642, China South China Agricultural University Guangzhou China; 2 Sichuan Cave Exploration Team, No. 66, 5th Shuangcheng Road, Chenghua District, Chengdu, 610051, China Sichuan Cave Exploration Team Chengdu China

**Keywords:** Carabids, new combination, new genera, new species, subterranean, taxonomy

## Abstract

Two new genera and four new species of cave-adapted ground beetles are described from Sichuan Province, southwestern China. *Uenoaphaenops***gen. nov.** is established to place the trechine species *Qianotrechus fani* Uéno, 2003 occurring in the limestone cave Hua’er Dong, southeastern Sichuan (Luzhou: Gulin). *Chu pheggomisetoides***gen. nov. & sp. nov.**, from the limestone cave Hanwang Dong, northeastern Sichuan (Guangyuan: Chaotian), is somewhat like the European cavernicolous trechine genus *Pheggomisetes* Knirsch, 1923, from Bulgaria and Serbia, in particular in the configurations of head and pronotum. *Boreaphaenops liyuani***sp. nov.**, also from Hanwang Dong, is the second representative of the genus and the first record in Sichuan. *Agonotrechus sinotroglophilus* Deuve, 1999, a troglophile, is reported from Sichuan for the first time. The other two new species belong to the platynine genus *Jujiroa* Uéno, 1952: *J. uenoi***sp. nov.** from the cave Banche Dong on the northern side of the Dadu River (Leshan: Shawan) and *J. wangzheni***sp. nov.** from the cave Hua’er Dong, which is sympatric with *Uenoaphaenops fani* (Uéno, 2003) **comb. nov.** A distribution map for the localities of all abovementioned caves and a key to *Jujiroa* species known in Sichuan are provided.

## Introduction

Sichuan Province without question holds the richest specific diversity of Carabidae*sensu lato* in China. Over one fourth (1189) of the total species (3946) known in China occur in Sichuan (Anichtchenko et al. 2007–2020). On the contrary, the subterranean fauna of Carabidae is comparatively poor in this province, with the only exception, the troglobitic platynine genus *Jujiroa* Uéno, 1952 which is very rich in Sichuan. Five of the eight *Jujiroa* species known in mainland China were reported from Sichuan (Vigna Taglianti 1995; Uéno and Kishimoto 2001; Uéno 2007; Deuve and Pütz 2013; Tian and He 2020).

However, the cave fauna of the ground beetles in Sichuan is interesting. For example, *Troglopatrobus zhouchaoi*Deuve et al., 2020, known only from the cave Lianhua Dong in Pengzhou in the northern suburb of Chengdu, the provincial capital city, is morphologically highly modified and the only Patrobini species occurring in subterranean habitats in the world (Deuve et al. 2020). Species of the genus *Sichuanotrechus* Deuve, 2005, together with Duvalioblemus (Shublemus) liyuaniDeuve et al., 2020 occur only in the Longmen Mountains of northern Sichuan (Deuve 2005; Uéno 2006, 2008; Huang and Tian 2015). *Qianotrechus fani* Uéno, 2003 is only found inside the cave Hua’er Dong in Gulin County, in the southeastern corner of the province (Uéno 2003; Deuve et al. 2020). *Agonotrechus sinotroglophilus* Deuve, 1999, a troglophilous species formerly recorded from Chongqing (Deuve 1999; Deuve and Tian 2016) was newly found in a cave in northeastern Sichuan. Another troglophilous species, *Trechiotes perroti* Jeannel, 1954, occurs in a large area in southwestern China including Sichuan (Deuve et al. 1999; Deuve and Tian 2011, 2016).

**Figure 1.**
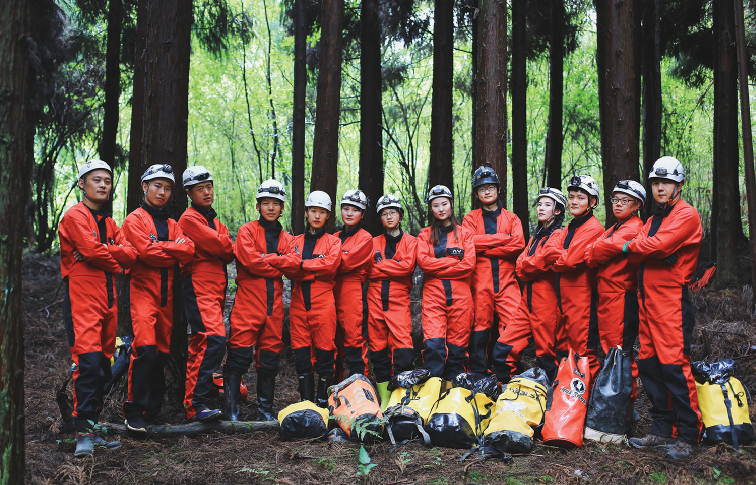
Group photo of some members of the Sichuan Cave Exploration Team (SCET, Chengdu).

Thanks to the Sichuan Cave Exploration Team (SCET, Chengdu), in which the cave biology group is led by Li He (the second author of the present paper), our knowledge of cave ground beetles in Sichuan Province is quickly increasing. The majority of members of SCET are young and active cavers (Fig. 1). In recent years, they have conducted many cave surveys which resulted in important scientific discoveries in terms of cave invertebrates, especially ground beetles. For instance, they found one of the richest cave fauna of ground beetles recorded from China in the limestone cave Hanwang Dong, northeastern Sichuan (Guangyuan: Chaotian). Four cave-adapted carabid species in total have been collected in this beautiful limestone cave: a pterostichine species belonging to the Pterostichus subgenus Huaius (Tian & He, 2020), two new troglobitic trechine species and the troglophile *Agonotrechus sinotroglophilus* Deuve, 1999. They have re-discovered almost all species of the genus *Sichuantrechus*. From two caves in Leshan and Luzhou respectively, they found another two new *Jujiroa* species apart from *J. deliciola* Uéno & Kishimoto, 2001 and the recently described *J. zhouchaoi* Tian & He, 2020 (Tian and He 2020).

*Qianotrechus fani* Uéno, 2003 was the first troglobitic trechine beetle reported from Sichuan. This species was named in honour of the person who provided crucial support to Dr Shun-Ichi Uéno during his collecting travels in China from 1998 to 2010 based on a so-called international scientific collaborative project, which resulted in fruitful discoveries on subterranean ground beetles. *Qianotrechus fani* is a very peculiar member within the genus *Qianotrechus* Uéno, 2000 not only because of its locality which is far from those of other congeners, but also its morphological character states which are very different from the other species of the genus. Uéno (2003) tentatively treated it as a *Qianotrechus* species because only a female was available at that time. Thanks to Dr Yunchun Li (an expert of Pseudoscorpiones from China West Normal University, Nanchong), the first author received three individuals of *Q. fani* collected in the cave Hua’er Dong, the type locality. Later, Yuan Li and Zhen Wang (both are local amateur entomologists) who also surveyed in Hua’er Dong, successfully collected three specimens of the species. Further laboratory study revealed that there is no sexual dimorphism in this species, i.e., the protarsi are not modified in male, and abdominal ventrite VII is quadrisetose in both sexes. Furthermore, the male genital organ of *Q. fani* is very short and stout, contrary to the other *Qianotrechus* species in which it is always thin and long. These crucial features, plus other peculiar morphological characters, strongly support that *Q. fani* belongs to an unknown genus rather than *Qianotrechus*.

The aim of this paper is to establish a new genus for Uéno’s *Qianotrechus fani*, describe another new genus and two new species of the tribe Trechini from the cave Hanwang Dong, give the first record of *Agonotrechus sinotroglophilus* for Sichuan Province, and describe two new species of the Platynini genus *Jujiroa*.

This paper is dedicated to the late Dr Shun-Ichi Uéno, a well-known cave biologist in National Science Museum (Natural History), Tokyo, who unfortunately passed away on October 3, 2020.

## Material and methods

**Figure 2.**
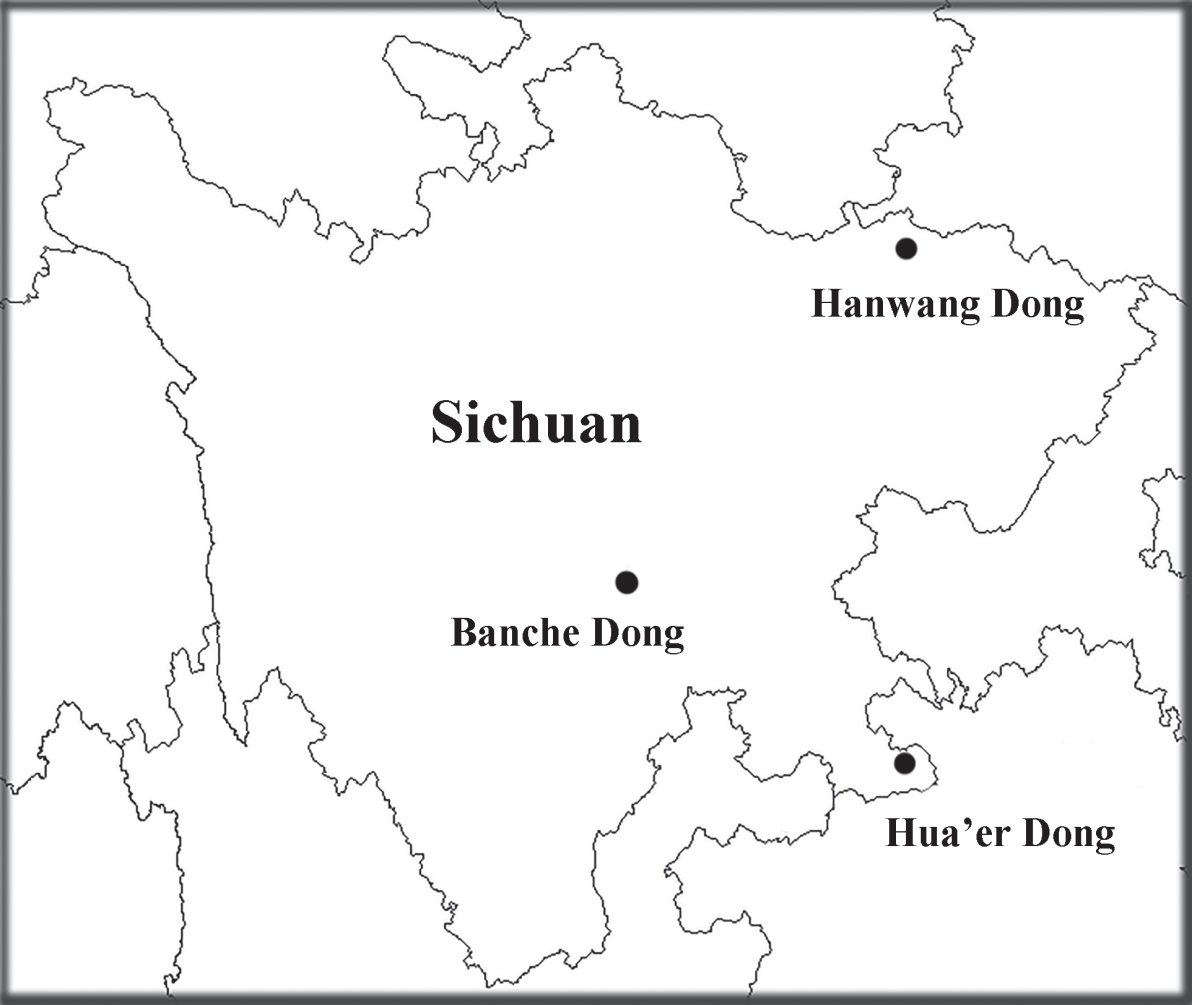
Map of Sichuan Province showing the locations of the related caves.

The material for this study were discovered from three caves in Sichuan Province, namely, Hanwang Dong, Hua’er Dong and Banche Dong (Fig. 2). Beetles were collected using an aspirator or trap baited with silkworm (*Bombyx mori* L., 1758) chrysalis meal, killed with ethyl acetate and kept in vials with 75% ethanol; a few specimens were kept in 95% ethanol, for DNA sequencing.

The specimens were examined with a Nikon SMZ1000 stereo-microscope in a solution of glycerin. All illustrations were completed using Adobe Illustrator CS 6.0 based on digital photos taken by means of a Keyence VHX-5000 digital microscope. The distribution map was drawn using MapInfo Professional 12.0 software.

Length of body is measured from the right mandible (when opened) to the apex of the elytra. Width of body is the maximum width of combined elytra. Abbreviations of measurements used in the text are as follows:

**EL** length of elytra, from base of scutellum to elytral apex

**EW** maximum width of combined elytra

**HLl** length of head excluding mandibles, from front of labrum to base of head

**HLm** length of head including mandibles, from apex of right mandible to base of head

**HW** maximum width of head

**PfW** width of pronotum at front

**PbW** width of pronotum at base

**PL** length of pronotum, through mid-line

**PW** maximum width of pronotum

The material examined for this study is deposited in the following collections:

**CLH** Collection of Li He, Chengdu, Sichuan, China

**CYL** Collection of Yuan Li, Deyang, Sichuan, China

**CZW** Collection of Zhen Wang, Chengdu, Sichuan, China

**SCAU** South China Agricultural University, Guangzhou, China

## Taxonomy

### Tribe Trechini Bonelli, 1810

#### Uenoaphaenops gen. nov.

Taxon classificationAnimaliaColeopteraCarabidae

EE95CFE4-E6B5-5F04-8E3E-F4D17ED7ECE9

http://zoobank.org/20CF5C96-DFC5-419F-97D8-924D18433C3F

**Figure 3.**
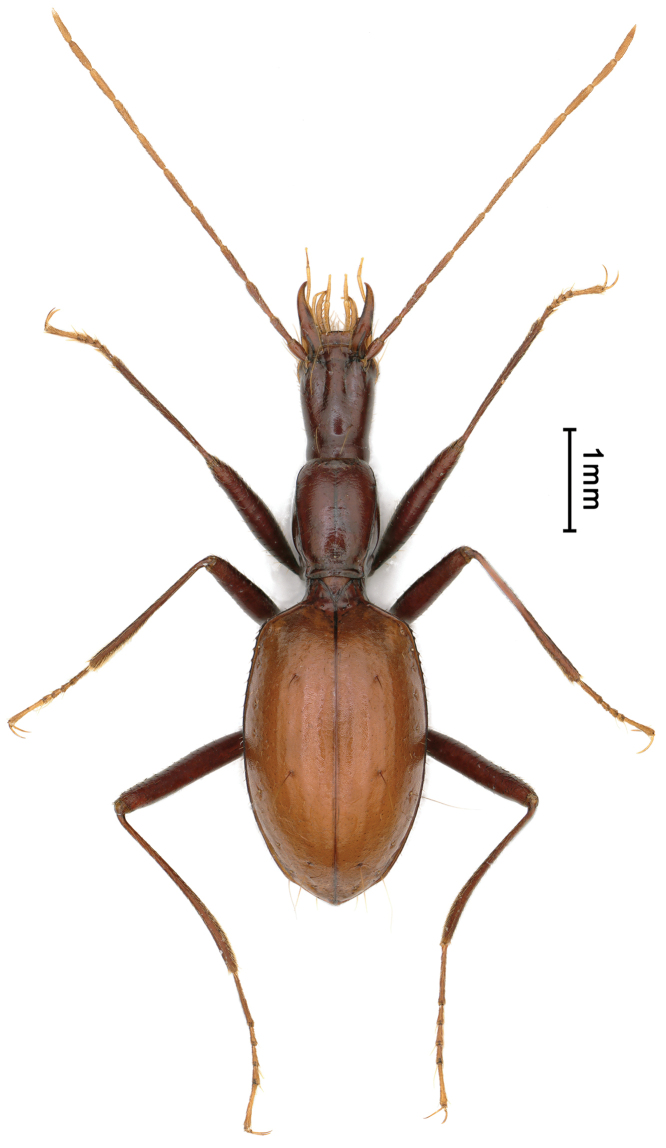
Habitus of *Uenoaphaenops fani* (Uéno, 2003), comb. nov., female.

**Figure 4.**
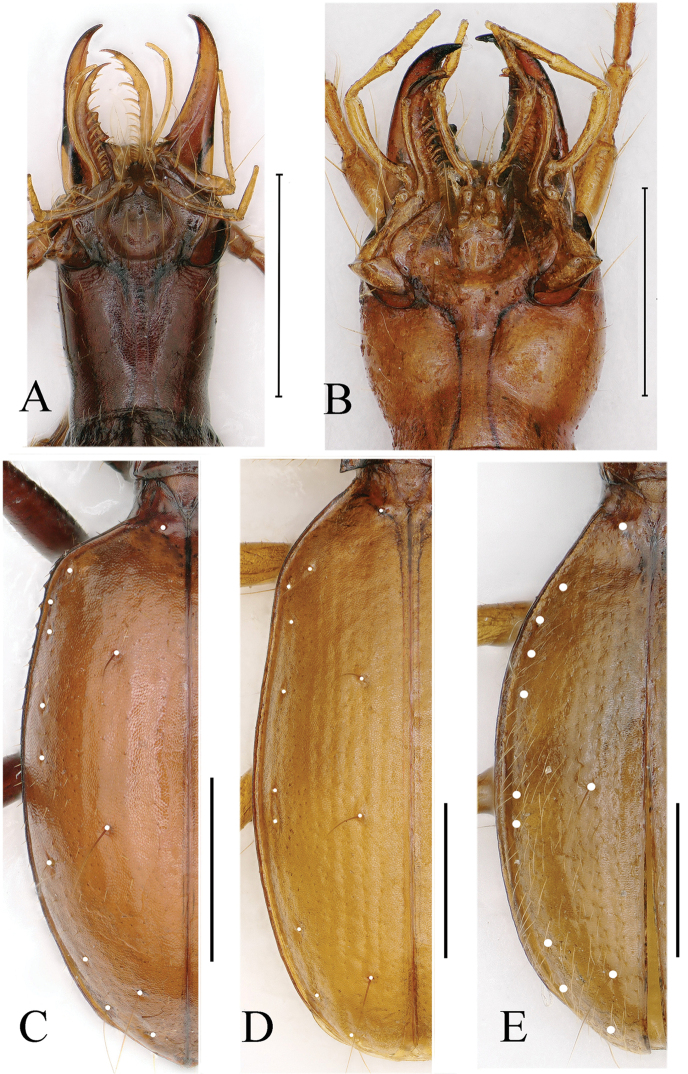
Cave trechine beetles **A** ventral head of *Uenoaphaenops fani* (Uéno, 2003), comb. nov., female **B** ventral head of *Chu pheggomisetoides* gen. nov. & sp. nov., female **C–E** elytral chaetotaxy of *Uenoaphaenops fani* (Uéno, 2003), comb. nov., female, *Chu pheggomisetoides* gen. nov. & sp. nov., female paratype, and *Boreaphaenops liyuani* sp. nov., female holotype. Scale bars: 1.0 mm.

**Figure 5.**
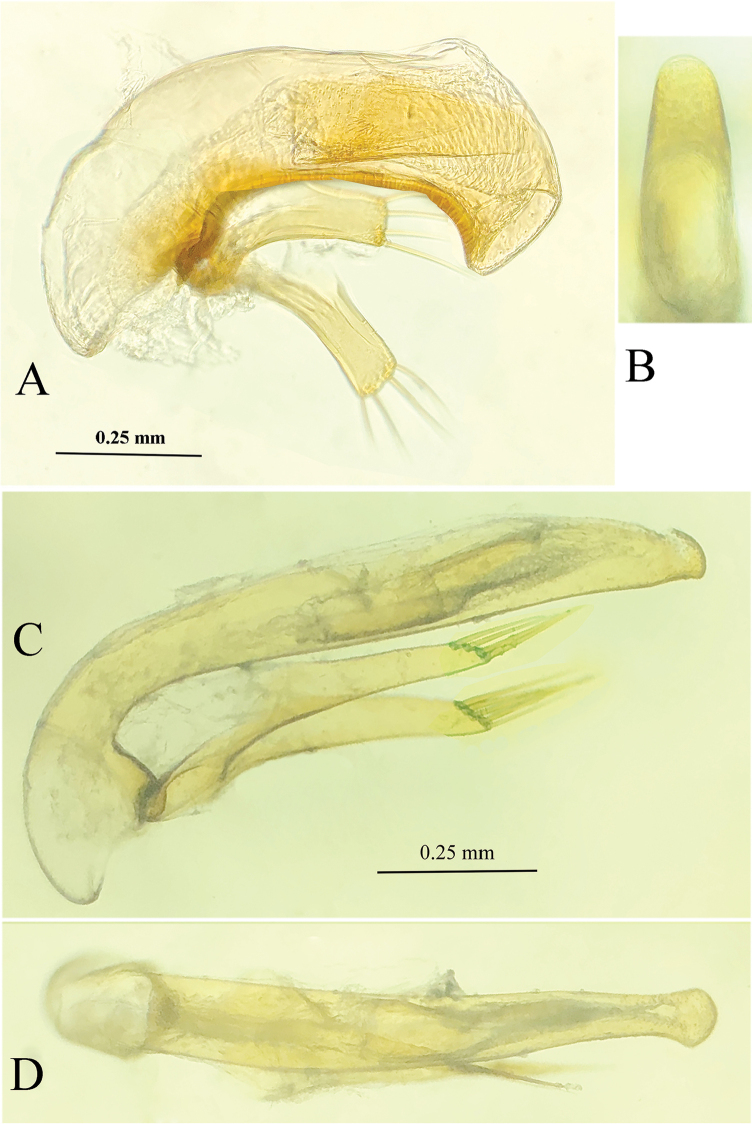
Male genitalia of cave trechine species, lateral and dorsal views **A, B***Uenoaphaenops fani* (Uéno, 2003), comb. nov. **C, D***Chu pheggomisetoides* gen. nov. & sp. nov.

Chinese name: 上野穴步甲属 Figs 2 , 3 , 4A, C , 5A, B

##### Type species.

*Qianotrechus fani* Uéno, 2003 from the cave Hua’er Dong, Gulin, southeastern Sichuan).

##### Generic characteristics.

Medium-sized, aphaenopsian and depigmented; body moderately elongate, wholly pubescent. Head strongly elongate, much longer than wide, nearly parallel-sided; neck weakly-marked, ring-shaped; two pairs of supraorbital setiferous pores present; frontal furrows incomplete, parallel-sided in most part though briefly divergent posteriorly; frons and vertex convex; right mandibular tooth bidentate; mentum and submentum completely fused; mentum bisetose, and covered with short setae on basal area of mental tooth, and along the site of labial suture; base largely concave, uni-foveate, tooth short and simple at tip; submentum with a row of 12 setae; labial palpomere 2 much longer than 3 (1.3 times), bisetose on inner margin, without additional setae; maxillary palpomere 3 much longer than 4 (1.4 times) (Fig. 4A); antennae long and thin, extending to about 1/4 of elytra from apex; one pair of suborbital pores present. Prothorax distinctly expanded, propleura visible from above; pronotum elongate, as long as head excluding mandibles, wider than head, widest at about 1/3 from front, lateral margins sinuate before hind angles which is nearly rectangular, base slightly narrower than front; two pairs of latero-marginal setae present, disc moderately convex. Elytra ovate, shoulders obtuse, almost rounded, distinctly serrate at prehumeral part, while ciliate on other parts; disc extraordinarily convex though depressed near base; striae reduced though traceable; presence of two dorsal pores along the 3^rd^ striae and the preapical; prehumeral set of the marginal umbilicate pores not aggregated, the 5^th^ pore much closer to 4^th^ than to 6^th^. Protarsi not modified in male; tibiae without longitudinal sulci. Ventrites IV–VI each with pair of paramedian setae; ventrite VII quadrisetose in both sexes.

**Male genitalia** (Fig. 5A, B). Aedeagus very short and small, but thick, weakly sclerotized. The median lobe slightly arcuate at median portion, but strongly sinuate before apex which is obtuse, with a large round opening; base quite large, without a sagittal aileron; inner sac provided with a thick and long copulatory piece, which is about 2/5 as long as aedeagus; in dorsal view, apical lobe gradually contracted towards apex which is broadly rounded; parameres well-developed, but much shorter than the median lobe, truncate at apical margin, each armed with four long setae at apex.

##### Remarks.

This peculiar species was put into the genus *Qianotrechus* Uéno, 2000 due to the lack of a male at that time. However, Uéno (2003) pointed out the following characteristics of this species which are not present in the congeners of *Qianotrechus*: body wholly pubescent; humeral margins of elytra strongly serrated; and the 5^th^ pore of marginal umbilicate series forwardly and inwardly shifted, widely distant from the 6^th^ pore. Hence, he mentioned that the above peculiarities may suggest a generic separation of this species from the Guizhou genus. Our examination of male individuals provided further evidence to support his opinion. First, protarsi are not modified in the male of *Qianotrechus fani*, while the 1^st^ and 2^nd^ protarsomeres are spurred inwards at the apices in all other *Qianotrechus*. Second, ventrite VII is quadrisetose in both sexes in *Qianotrechus fani*, vs. bisetose in males of other *Qianotrechus*. Third, the aedeagus of *Qianotrechus fani* is very small and stout, not the same type as in other *Qianotrechus* species, which are always large and elongate (Uéno 2000, 2003).

##### Etymology.

“*Ueno*”+ “-*aphaenops*”. Dedicated to the late Dr Shun-Ichi Uéno who made a great contribution to the knowledge of Chinese subterranean ground beetles. Gender masculine.

##### Generic range.

China (Sichuan) (Fig. 2). A monospecific genus only recorded from the cave Hua’er Dong, Gulin County, southeastern Sichuan.

#### Uenoaphaenops fani

Taxon classificationAnimaliaColeopteraCarabidae

(Uéno, 2003) comb. nov.

5B3EF876-46B6-594C-B2C0-2108D3EBC33B

**Figure 6.**
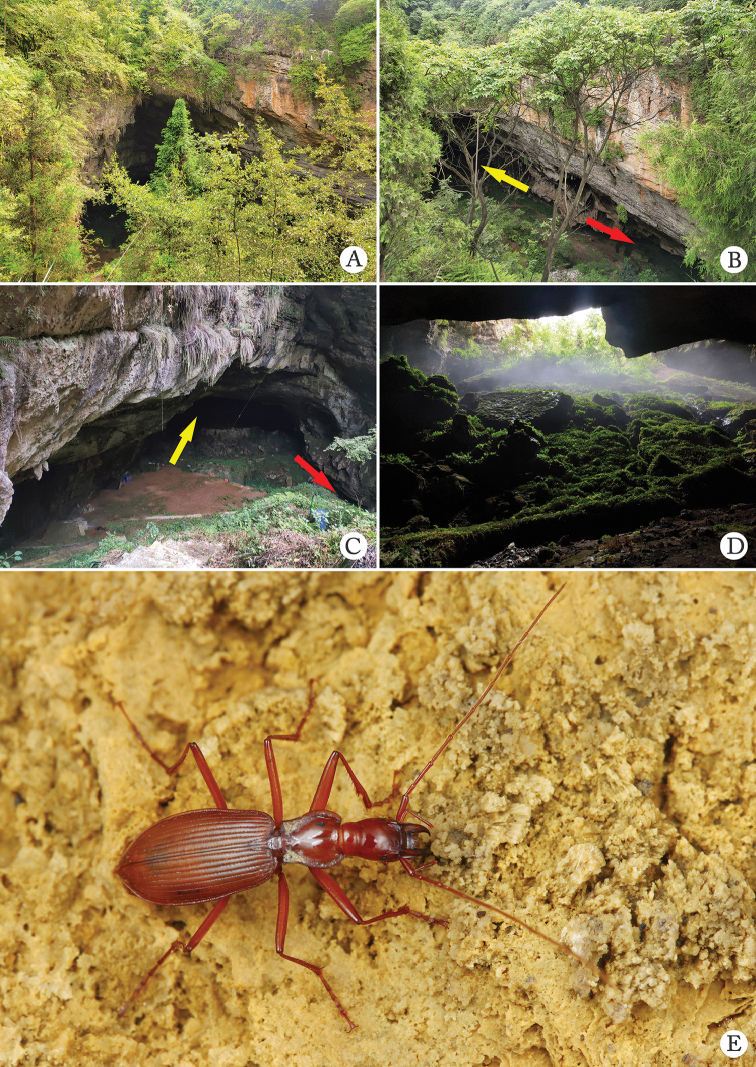
Cave Hua’er Dong, the type locality of *Uenoaphaenops fani* (Uéno, 2003), comb. nov. and *Jujiroa wangzheni* sp. nov. **A–C** environs of the cave, left (yellow arrow) and right (red arrow) entrances **D** entrance to the right **E** a running individual of *Jujiroa wangzheni* sp. nov. in cave. (**A–D** by courtesy of Yuan Li).

Chinese name: 范氏上野穴步甲 Figs 2 , 3 , 4A, C , 5A, B , 6

##### Material.

3 females, the cave Hua’er Dong, Xiangdingshan, Xiangding, Shiping, Gulin, Luzhou, Sichuan (四川省泸州市古蔺县石屏镇向顶村象顶山华儿洞), 28.028931°N, 106.00716°E, 640 m, 2020-VI-22, leg. Yuan Li & Zhen Wang, in CLH, CYL and CZW, respectively; 2 males and 1 female, same cave, 2019-XI-3, leg. Yunchun Li, in SCAU.

##### Remarks.

Uéno (2003) mentioned that the submentum of *Qianotrechus fani* is 8-setose. Actually, there are 12 setae in total in our exemplars, excluding the shorter pubescence along the site of labial suture.

##### Distribution.

China (Sichuan). Known only from the cave Hua’er Dong (Figs 2, 6).

Uéno (2003) gave a detailed description of the cave Hua’er Dong in which there are two main entrances in opposite directions (Fig. 6A–D). The exemplars of *Uenoaphaenops fani* (Uéno, 2003) comb. nov. were collected on the wall or under stone in the moist areas about 20–30 m from the left entrance in dark zone. It is sympatric with *Jujiroa wangzheni* sp. nov. (Fig. 6E).

#### Chu gen. nov.

Taxon classificationAnimaliaColeopteraCarabidae

6400A3E5-E788-56DC-B477-D642C5739991

http://zoobank.org/323F87B4-17A4-48F4-B816-8423C9011A68

**Figure 7.**
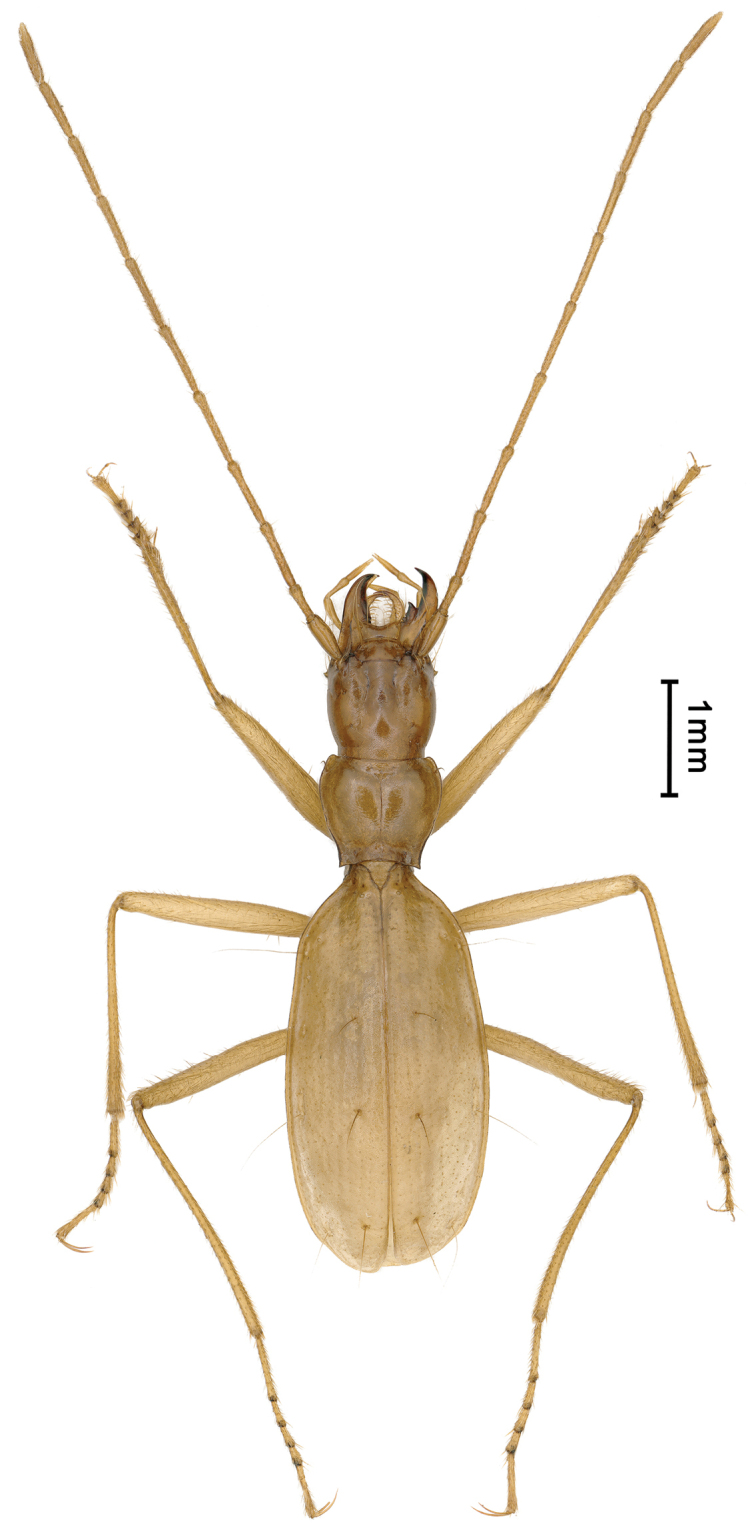
Habitus of *Chu pheggomisetoides* gen. nov. & sp. nov., male holotype.

**Figure 8.**
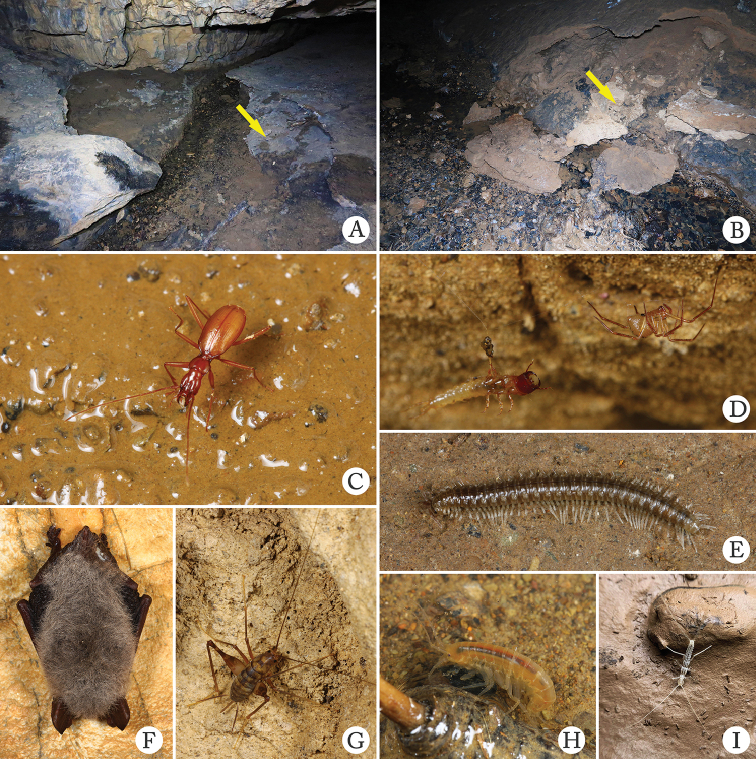
Cave Hanwang Dong, the type locality of *Chu pheggomisetoides* gen. nov. & sp. nov. and *Boreaphaenops liyuani* sp. nov., and some sympatric cave animals **A, B** habitat inside the cave, arrow in **A** indicates the place where a *Chu pheggomisetoides* gen. nov. & sp. nov. was found, arrow in **B** indicates the place where the single female of *Boreaphaenops liyuani* sp. nov. was found **C** an individual of *Chu pheggomisetoides* gen. nov. & sp. nov. running in cave **D** a ground beetle’s larva was trapped in web of spider *Pseudonesticus* sp. **E** a *Nepalella* millipede **F** a bat **G** a cricket **H***Gammarus qinling* Hou & Li, 2018 **I** a dipluran.

Chinese name: 初盲步甲属 Figs 2 , 4B, D , 5C, D , 7 , 8

##### Type species.

*Chu pheggomisetoides* sp. nov., from the limestone cave Hanwang Dong in Guangyuan, northeastern Sichuan.

##### Generic characteristics.

Medium-sized, somewhat similar to the Balkan genus *Pheggomisetes* Knirsch, 1923 in appearance especially head and pronotum (Fig. 7); anophthalmic and depigmented; body moderately elongate, with rather thin and slender appendages. Head strongly expanded laterally and convex though shorter than long, two pairs of supraorbital setiferous pores present; frontal furrows long and well-marked; labrum widely emarginated at front, mandibles widened and developed, apices strongly hooked, right mandibular tooth tridentate; labial suture visible at side, completely disappeared medially (Fig. 4B); mentum bisetose, base largely concave, submentum 8-setose; antennae thin and very long, extending over apices of elytra. Propleura invisible from above; pronotum subcordate, transverse, hind angles very sharp and distinctly protruded backwardly which is similar in *Pheggomisetes*, only presence of the anterior pair of latero-marginal setae. Elytra elongated ovate, twice as long as wide, much longer than fore body; prehumeral angles rounded off; lateral margins well-bordered throughout, finely ciliate on shoulders, whereas smooth on other parts; disc moderately convex, striae noticeable though distinctly reduced; two pairs of dorsal and the preapical setiferous pores present; the humeral group of the marginal umbilicate pores not aggregated, the 1^st^ pore inwardly shifted to the site of 7^th^ stria, only the 2^nd^ pore closest to marginal gutter, 4^th^ shifted posteriad; 5^th^ and 6^th^ pores closely spaced (Fig. 4D). The 1^st^ and 2^nd^ protarsomeres modified in male, distinctly widened and bluntly denticulate inward at apices, and with spongy setae ventrally. Ventrite VII with one pair of apical setae in male, whereas two pairs in female. Male genitalia with median lobe almost straight, suddenly widened at apical part; in lateral view, apex notched dorsally (Fig. 5C, D).

##### Remarks.

Although *Chu* gen. nov. resembles the Balkan cavernicolous genus *Pheggomisetes* Knirsch, 1923 (Knirsch 1923; Vrbica et al. 2017) in the shape of head and pronotum, this must be a case of convergence because the faunas in the Balkan Peninsula and China are completely different. Indeed, there are many cases of large gaps in cave trechines between Europe and China, e.g., *Doderotrechus* from Western Italy vs. *Trechus* of the *dacaitranus* group (*Trechus bastropi*) from Tibet (Faille et al. 2013). Apart from the similarities of the head and pronotum, both *Chu* and *Pheggomisetes* are very different in many aspects including outlines of head, prothorax and elytra, and in particular the chaetotaxic pattern on head and elytra.

As the fauna of subterranean ground beetles are still not well known, the phylogenetic position of *Chu* gen. nov. among Trechini is unknown at present, like many other genera. Its peculiar characters, such as ellipsoidal head with deep emarginated labrum, stout mandible, subcordate pronotum with sharp and backwardly protruded hind angles, and almost straight median lobe which is enlarged and notched at the apex isolate *Chu* gen. nov. from all other Chinese trechines. Beside comparative morphological study, evidence from molecular analysis would be valuable in determining its position.

##### Etymology.

“*Chu*” means “the first time” in Chinese, suggesting that this species was the first subterranean beetle discovered by Li He (the second author). Gender masculine.

##### Generic range.

China (Sichuan) (Fig. 2). Monospecific genus, known only from the cave Hanwang Dong in Chaotian, Guangyuan.

#### Chu pheggomisetoides sp. nov.

Taxon classificationAnimaliaColeopteraCarabidae

66DEE8DB-2370-557A-9E48-C7CA23B5F5C6

http://zoobank.org/5CB92BFD-836E-4342-8ADD-70DA2C6C4A81

Chinese name: 汉王初盲步甲 Figs 2 , 4B, D , 5C, D , 7 , 8

##### Material.

***Holotype***: male, cave Hanwang Dong, Zhongbai, Zengjia, Chaotian, Guangyuan, Sichuan (四川省广元市朝天区曾家镇中柏村汉王洞), 32.577297°N, 106.106979°E, 1210 m, 2020-VI-07, leg. Li He & Yuan Li, in SCAU. ***Paratypes***: 1 male, *idem*, in SCAU; 1 female, same cave as above, 2020-VI-06, leg. Li He, Yuan Li & Yimei Wen, in CLH; 1 female, same cave, 2018-IV-06, leg. Li He, in SCAU.

##### Diagnosis.

Medium-sized troglobitic beetles, eyeless and lacking pigmentation, somewhat similar to a *Pheggomisetes* species of Bulgaria and Serbia in Balkan Peninsula due to its convex head and subcordate pronotum with very sharpened hind angles.

##### Description.

***Length***: 6.0–6.5 mm; width: 1.5 mm. Habitus as in Fig. 7.

***Body*** yellow or brown, but antennae, palps and tarsi paler; surface glabrous and smooth though genae sparsely setose and elytra covered with a few, minute, pubescence on lateral margins. Underside of head with a few sparse setae, of thorax and abdominal ventrites glabrous. Microsculpture engraved meshes more or less polygonal on head and pronotum, and irregularly and densely striate on elytra.

***Head*** slightly elongate, ellipsoidal, longer than wide, HLm/HW = 1.67–1.72, HLl/HW = 1.18–1.28; genae expanded laterally, widest a little behind middle of head excluding mandibles, neck constriction broad, moderately defined, frons and vertex strongly convex, anterior and posterior supraorbital setiferous pores narrowly spaced, frontal furrows deep and fairly long, strongly divergent posteriorly, ending beside posterior supraorbital pores; clypeus quadrisetose; labrum transverse, deeply and widely emarginated at frontal margin, 6-setose; mandible stout and widened, curved at apical 1/3, strongly hooked at apices, right mandibular tooth very developed; labial suture disappearing medially, making mentum and submentum partly fused; mentum bisetose, base largely concave, tooth short and bifid at apex, about half as long as lateral lobes; submentum 8-setose; ligula 6-setose at apex; palps moderately elongate and glabrous but the 2^nd^ labial palpomere bisetose on inner margin, with an additional seta at outer margin at subapex; 2^nd^ labial palpomere slightly longer than 3^rd^; 3^rd^ maxillary palpomere as long as 4^th^; suborbital pores located on ventral side of head, intermedial between neck constriction and submentum (Fig. 4B). Antennae with 10^th^ and 11^th^ antennomeres extending over elytral apices, 1^st^ antennomere smooth and stout, covered several long setae, pubescent from the 2^nd^ antennomere; relative length of each antennomere compared with the 2^nd^ in the holotype as: the 1^st^ (1.13), 2^nd^ (1.00), 3^rd^ (1.67), 4^th^ (1.73), 5^th^ (1.93), 6^th^ (1.87), 7^th^ (1.80), 8^th^ (1.57), 9^th^ (1.54), 10^th^ (1.43) and 11^th^ (1.57).

***Pronotum*** wider than long, PnL /PnW = 0.76–0.83; much shorter than head without mandibles, PnL/HLl = 0.55–0.75; wider than head, PnW/HW = 1.07–1.11; lateral margins and front finely bordered, widest at about 1/3 from front, gently narrowed anteriorly and posteriorly, but strongly curved before base, forming a large and acute hind angle with the arcuate base; anterior latero-marginal setae at about 1/7 from front; front slightly emarginate, distinctly wider than base, Pnb/Pnf = 0.83–0.86; disc moderately convex, mid-line clear, both front and posterior transversal impressions moderately marked. Scutellum large.

***Elytra*** much longer than fore body including mandibles, much longer than wide, EL/EW = 1.90–2.02; much wider than pronotum, EW/PrW = 1.65–1.67; base unbordered, prehumeral part widely rounded, lateral margins finely but well-bordered throughout, widest at about middle, gently and gradually contracted towards base and apices; disc moderately convex though slightly depressed on each elytron near base; striae faint but noticeable; basal pore present at side but behind of scutellum, anterior and posterior dorsal pores along the 3^rd^ stria located at about basal 1/5 and apical 2/5 of elytra respectively, preapical pore at about apical 1/8 of elytra, much closer to suture than to apical margin; locations of the marginal umbilicate pores as in Fig. 4D.

***Legs*** densely pubescent; the 1^st^ tarsomere much, and slightly shorter than 2^nd^–4^th^ combined in fore and middle legs, respectively, whereas as long as in hind ones; tibiae without longitudinal sulci.

***Ventrites*** pubescent; IV–V each with two pairs, VI with three pairs of paramedian setae, and several additional setae which are much shorter; VII bisetose in male, while quadrisetose in female.

**Male genitalia** (Fig. 5C, D). Median lobe and parameres long and thin, suddenly curved at basal one fourth, then nearly straight towards apex. Basal opening small, without a sagittal aileron; inner sac provided with a long copulatory piece, which is about 1/3 as long as aedeagus; in dorsal view, apical lobe suddenly narrowed before the enlarged apex which is broadly rounded; each paramere armed with four long setae at apex.

##### Etymology.

Refers to the similarity of this new species with a *Pheggomisetes* species from Balkans.

##### Distribution.

China (Sichuan). Known only from the cave Hanwang Dong in Guangyuan (Fig. 2), sympatric with *Boreaphaenops liyuani* sp. nov., *Agonotrechus sinotroglophilus* Deuve, 1999 and Pterostichus (Huaius) hanwang Tian & He, 2020.

The exemplars of *Chu pheggomisetoides* gen. nov. & sp. nov. were collected under a stone at the water edge in the innermost main passage in the cave (Fig. 8A–C). In addition to the four ground beetle species mentioned above, other animals found in Hanwang Dong are a *Pseudonesticus* spider, a *Nepalella* millipede, a *Gammarus* amphipod, bats, crickets and diplurans (Fig. 8D–I).

#### Boreaphaenops liyuani sp. nov.

Taxon classificationAnimaliaColeopteraCarabidae

E55DA15B-EFC1-57D5-BAA9-7E1D53878356

http://zoobank.org/7FA4C38E-65A2-42F1-BB95-12784F9AFAB9

**Figure 9.**
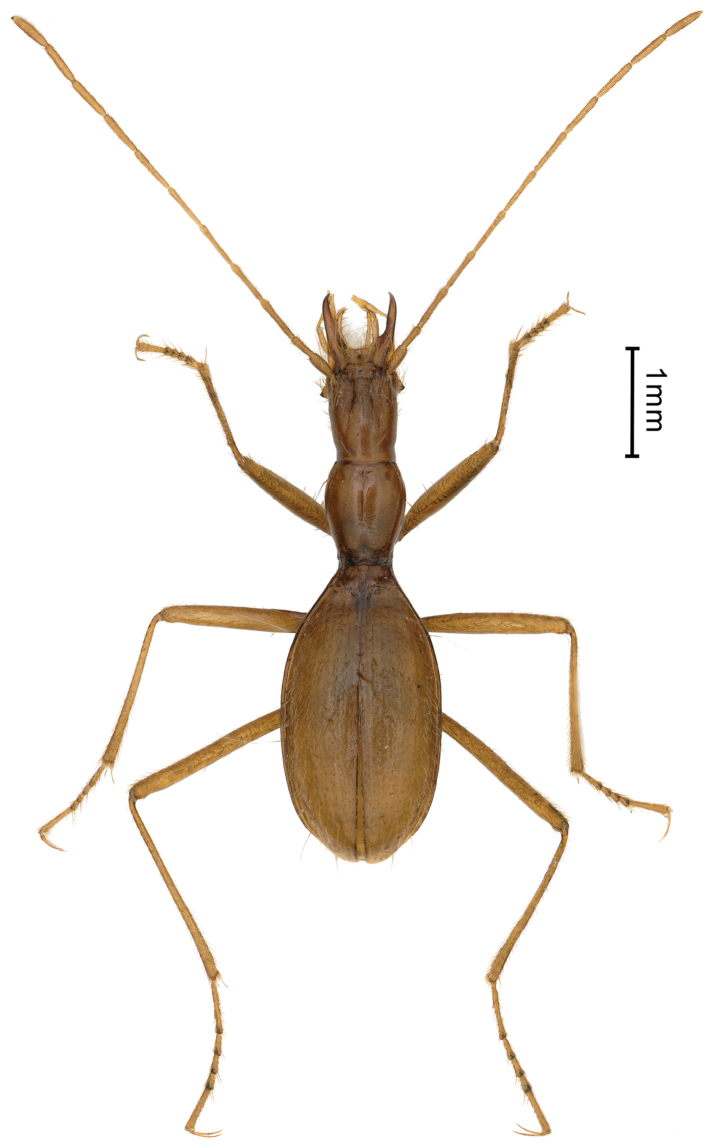
Habitus of *Boreaphaenops liyuani* sp. nov., female holotype.

**Figure 10.**
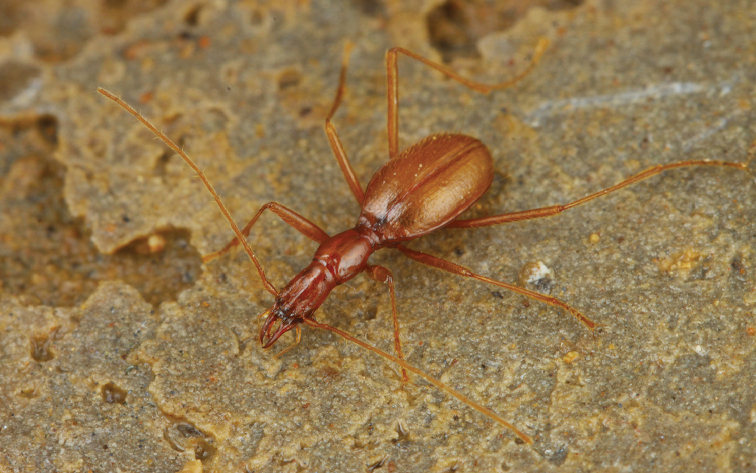
An individual of *Boreaphaenops liyuani* sp. nov. running in cave.

Chinese name: 李圆北盲步甲 Figs 2 , 4E , 9 , 10

##### Material.

***Holotype***: female, the cave Hanwang Dong, Zhongbai, Zengjia, Chaotian, Guangyuan, Sichuan (四川省广元市朝天区曾家镇中柏村汉王洞), 32.577297°N, 106.106979°E, 1210 m, 2020-VI-6, leg. Li He, Yuan Li & Yimei Wen, in SCAU.

##### Diagnosis.

An aphaenopsian, small-sized beetle, eyeless and depigmented, body distinctly elongate, with thin and long appendages, densely pubescent on head and elytra, presence of only a dorsal setiferous pore along the 3^rd^ stria on each elytron.

##### Description.

***Length***: 5.2 mm, width: 1.3 mm. Habitus as in Fig. 9.

***Body*** brown, but antennae, palps and tarsi paler; head and elytra covered with dense pubescence, pronotum glabrous. Underside of head with a few sparse setae, of thorax and abdominal ventrites glabrous. Microsculpture engraved meshes more or less isodiametric on head, strongly transverse on pronotum and densely striate on elytra.

***Head*** similar to *B. angustus* Uéno, 2002 from the cave Lenre Dong in Shenlongjia, western Hubei Province (Uéno 2002), but with only two pairs of supraorbital setiferous pores instead of three; elongate, much longer than wide, HLm/HW = 2.56, HLl/HW = 1.58; nearly parallel-sided due to genae not convex instead of slightly expanded; widest at about middle of head excluding mandibles, neck constriction well-marked; frons moderately, and vertex strongly convex respectively; anterior and posterior supraorbital pores located at middle and basal 2/9 of head excluding mandibles, frontal furrows strongly divergent, ended near posterior supraorbital pores; clypeus 6-setose; labrum transverse, straight at frontal margin, 6-setose; mandible thin and elongate, gently hooked apically, right mandibular tooth bidentate though distinctly reduced; labial suture completely disappeared; mentum tooth very small, shorter than half of the lateral lobes, bifid at tip, with two setae on each side of base; ligula adnated with paraglossae, 8-setose at apex; basal foveae large and separated; submentum 10-setose; palps thin, slender and glabrous, but bisetose on inner margin of the 2^nd^ labial palpomere which is very long and 1.70 times as long as 3^rd^; 3^rd^ maxillary palpomere 1.15 times as long as 4^th^; suborbital pores intermediate between neck and submentum; antennae pubescent from the 2^nd^ antennomere, 1^st^ antennomere stouter covered with several long setae, slightly longer than 2^nd^; 3^rd^ to 6^th^ longer, subequal to one another; relative length of each antennomere compared with the 2^nd^ in the holotype as: the 1^st^ (1.05), 2^nd^ (1.00), 3^rd^ (1.82), 4^th^ (1.82), 5^th^ (1.82), 6^th^ (1.82), 7^th^ (1.82), 8^th^ (1.64), 9^th^ (1.64), 10^th^ (1.46) and 11^th^ (1.27).

***Prothorax*** slightly tumid at sides, propleura medially visible from above, slightly wider than pronotum; pronotum similar in *B. angustus* but more elongate, fore angles distinct, hind ones nearly rectangular; much longer than wide, PnL/PnW = 1.32; slightly shorter than head without mandibles, PnL/HLl = 0.95; wider than head, PnW/HW = 1.19; widest at about 3/4 from base; lateral margins almost vanished at 1/4 portion from base, finely bordered in other parts; base and front nearly straight, unbordered, the former narrower than the latter, Pnb/Pnf = 0.69; anterior latero-marginal setae at about 1/6 from front, posterior ones before hind angles; disc moderately convex, mid-line clear, both front and posterior transversal impressions faintly marked. Scutellum small and elongated.

***Elytra*** longer than fore body including mandibles, much longer than wide, EL/EW = 1.94; nearly twice as wide as prothorax, EW/PrW = 1.96; base unbordered; similar in *B. angustus* but devoid of humeral angles, lateral margins finely but well-bordered throughout, smooth; widest at about middle, gently contracted towards base but strongly to apices; disc moderately convex though depressed near base just behind basal pores; striae faint but well-indicated from the 1^st^ to 4^th^; basal pore present at sides of scutellum, only a median dorsal pore present along the 3^rd^ stria at a little behind middle; preapical pore present at about apical 1/7 of elytra, much closer to suture than to apical margin; marginal umbilicate pores well-marked, 2^nd^ closer to marginal gutter than others, prehumeral set (1^st^ to 4^th^) equidistantly located (Fig. 4E).

***Legs*** densely pubescent; 1^st^ tarsomere much and slightly shorter than 2^nd^–4^th^ combined in fore and middle legs, whereas as long in hind ones; tibiae without longitudinal sulci. Abdominal ventrite IV–VI each with two pairs of paramedial setae, ventrite VII quadrisetose.

**Male.** Unknown.

##### Etymology.

In honour of Mr. Yuan Li (Deyang, Sichuan), a co-collector of the type material.

##### Remarks.

The cave Hanwang Dong is about 400 km in a straight line from Lengre Dong, the locality of *B. angustus* Uéno, 2002, though both localities belong to same range of the Daba-Micang Mountains. Although *B. liyuani* sp. nov. has several differences from *B. angustus* which are probably of generic importance, e.g. completely fused mentum and submentum (labial suture visible in the latter species), thin and straight mandibles with tooth distinctly reduced (well-developed in *B. angustus*), smooth elytral lateral margins (ciliate in *B. angustus*), and very long 2^nd^ labial palpomere which is 1.7 time as long as the 3^rd^ (such a feature never observed in other Chinese cave trechines), we prefer to describe it as a member of *Boreaphaenops* at present as only a single female exemplar is available. In addition, it also differs from *B. angustus* in having smaller body size, presence of only a pair of posterior supraorbital pores on the head instead of two, pronotum well-angulate on hind and fore angles instead of rounded, lack of prehumeral angles of elytra which have only a single dorsal setiferous pore along the 3^rd^ stria instead of three in *B. angustus*, and with equidistant prehumeral pores of the marginal umbilicate series, vs. 4^th^ pore far from 3^rd^ in *B. angustus*.

##### Distribution.

China (Sichuan). Known only from the cave Hanwang Dong in Guangyuan (Fig. 2).

*Boreaphaenops liyuani* sp. nov. is the first representative of the genus from Sichuan Province, living together with *Chu pheggomisetoides* gen. nov. & sp. nov., *Agonotrechus sinotroglophilus* Deuve, 1999 and Pterostichus (Huaius) hanwang Tian & He, 2020. It is very rare in the cave: three surveys carried out by SCET led to the discovery of only a single female, which was collected under a stone at the water edge in the innermost main passage in the cave (Fig. 10).

#### Agonotrechus sinotroglophilus

Taxon classificationAnimaliaColeopteraCarabidae

Deuve, 1999

05963270-3313-5820-8CA7-9F9F11CBCEB1

Figs 2 , 11

Deuve 1999: 152; Deuve and Tian 2016: 352

##### Material.

1 male, cave Hanwang Dong, Zhongbai, Zengjia, Chaotian, Guangyuan, Sichuan (四川省广元市朝天区曾家镇中柏村汉王洞), 32.577297°N, 106.106979°E, 1210 m, 2018-IV-06, leg. Li He, in SCAU; 1 female, same cave, 2020-VI-06, leg. Li He, Yuan Li & Yimei Wen, in CLH.

##### Diagnosis.

**Figure 11.**
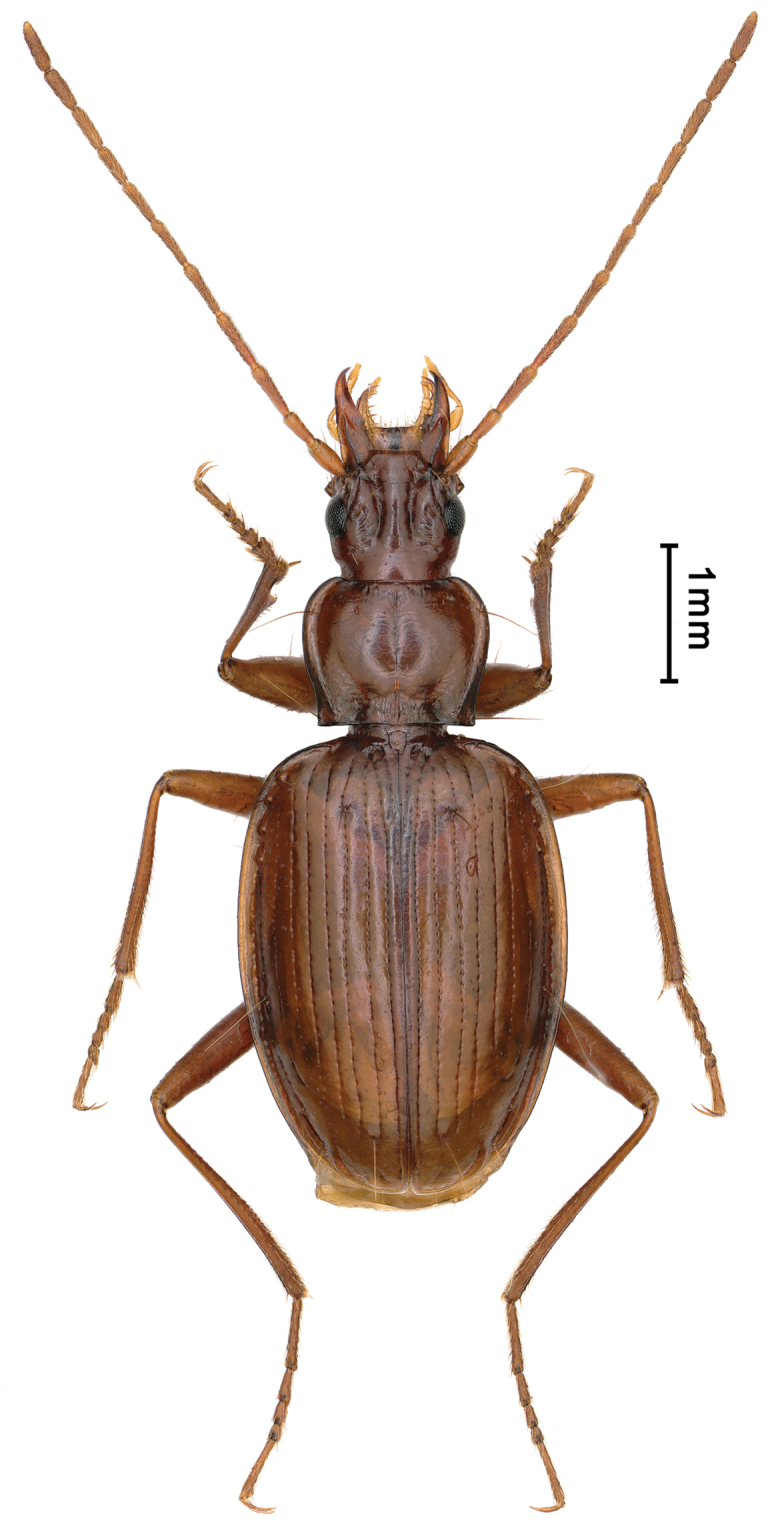
Habitus of *Agonotrechus sinotroglophilus* Deuve, 1999, male.

A troglophilous species though depigmented, macrophthalmic, body stout, with short appendages, developed frontal furrows on head, humeral set of the marginal umbilicate pores aggregated. Habitus as in Fig. 11.

##### Remarks.

This species was formerly reported from two limestone caves in Chongqing Municipality (Deuve 1999; Deuve and Tian 2016). This is the first record in Sichuan Province.

##### Distribution.

China (Chongqing and Sichuan) (Fig. 2).

The exemplars of *Agonotrechus sinotroglophilus* Deuve, 1999 were collected under stone in the moist area about 100–200 m from the entrance in Hanwang Dong.

### Tribe Platynini Bonelli, 1810

**Genus *Jujiroa* Uéno, 1952**

#### Jujiroa uenoi sp. nov.

Taxon classificationAnimaliaColeopteraCarabidae

9461DEBF-D1A3-570B-9150-4A4E874E6A7D

http://zoobank.org/B466CD2F-3ABC-47E0-8EF9-3B4B7344A305

**Figure 12.**
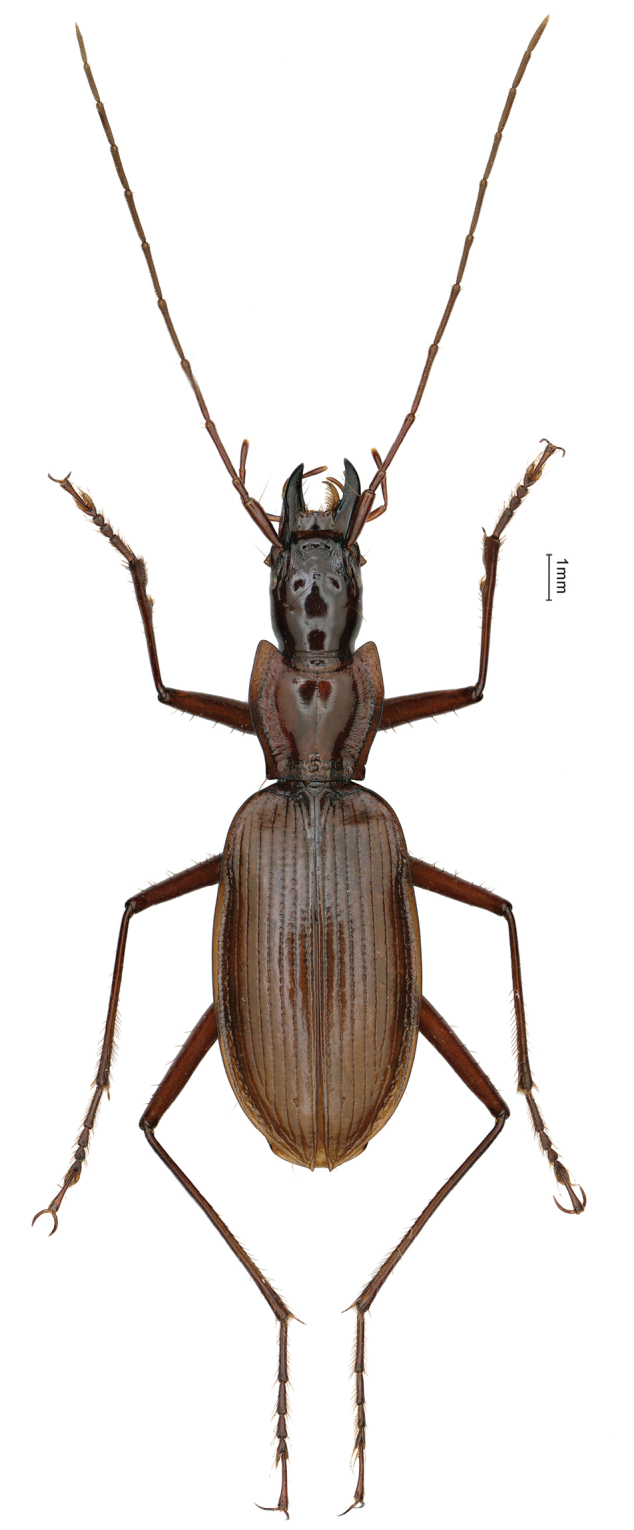
Habitus of *Jujiroa uenoi* sp. nov., male holotype.

**Figure 13.**
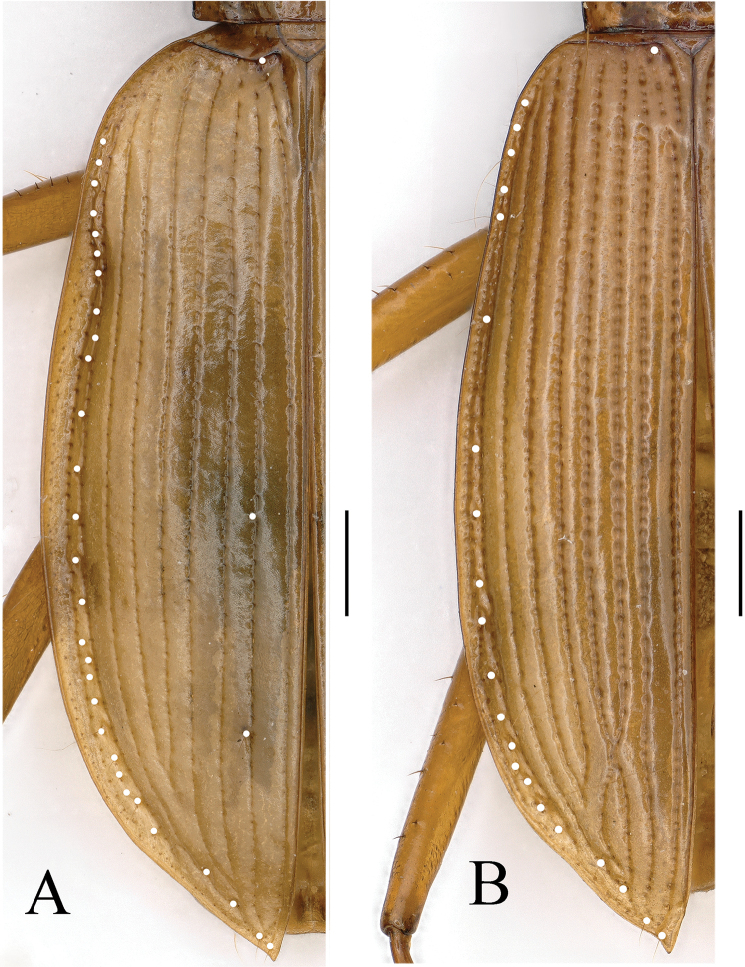
Elytral chaetotaxy of *Jujiroa* species **A***J. uenoi* sp. nov., male holotype **B***J. wangzheni* sp. nov., female holotype.

**Figure 14.**
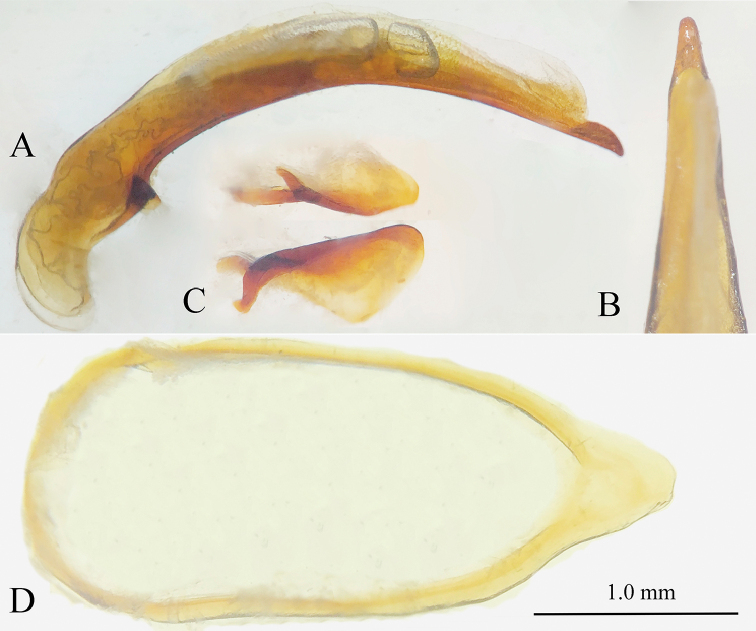
Male genitalia of *Jujiroa uenoi* sp. nov. **A** median lobe, lateral view **B** apical part of median lobe, dorsal view **C** parameres **D** genital ring, ventral view.

**Figure 15.**
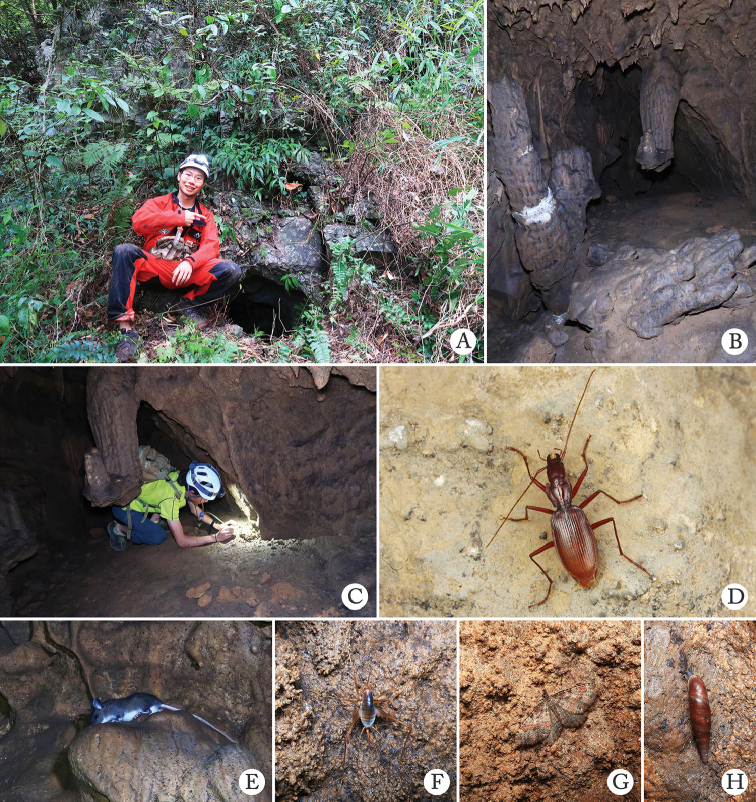
Cave Banche Dong, the type locality of *Jujiroa uenoi* sp. nov., and some sympatric cave animals **A** Li He in front of Banche Dong **B** environs inside cave, arrow indicating the place where a *J. uenoi* was found **C** Yuan Li is collecting **D** an individual of *J. uenoi* running in cave **E***Leopoldamys edwardsi* (Thomas, 1882) **F** a cricket **G** a moth **H***Serriphaedusa serrata* Deshayes, 1870.

Chinese name: 上野穴胫步甲 Figs 2 , 12 , 13A , 14 , 15

##### Material.

***Holotype***: male, cave Banche Dong, Jianshanzi, north side of the Dadu River, Shawan, Leshan, Sichuan (四川省乐山市沙湾区大渡河北岸尖山子搬车洞), 29.21043°N, 103.58349°E, 670 m, 2020-V-30, leg. Li He, Yuan Li & Hao Long, in SCAU. ***Paratype***: 1 female, *idem*, in SCAU.

##### Diagnosis.

Medium-sized *Jujiroa* species, body depigmented, microphthalmic, head thin and slightly expanded at sides, antennae not extending to apices of elytra, fore angles of pronotum distinctly protruded, elytra mucronate at apices, striae finely punctate, presence of two dorsal pores along the 2^nd^ stria, tarsi smooth.

##### Description.

***Length***: 15.0–15.5 mm; width: 4.5 mm. Habitus as in Fig. 12.

***Body*** concolorous yellow or brown, but a little darker on head, surface smooth and glabrous (though rough on sides and lateral margins of pronotum), moderately shiny. Microsculptural meshes finely and transversely striate on head, pronotum and elytra.

***Head*** thin and elongate, much longer than wide, HLm/HW = 2.06–2.11, HLl/HW = 1.53–1.48; widest just behind the level of eyes; genae convex, and slightly expanded at side; frontal furrows short and shallow, ending before the level of anterior supraorbital pores; two pairs of supraorbital pores present, anterior at about middle of head from labrum to neck, posterior about basal 2/7 of head; eyes very small and flat; clypeus bisetose, labrum bisinuate at front margin, 6-setose; mandibles elongated, teeth reduced; labial suture clear; mentum with two setae on each side just in front of the basal pits which are very small; median tooth short, about half as long as lateral lobes, bluntly bifid at tip; submentum with two setae on each side, inner ones longer; ligula short, widened and truncated at apical margin, bisetose; palpomeres long and slender, the 2^nd^ labial palpomere bisetose on inner margin, 1.2 times as long as 3^rd^, the 3^rd^ maxillary palpomere as long as 4^th^; antennae filiform, thin and very long, extended to apical 1/10 (male) or 1/9 (female) of elytra, the 1^st^ to 3^rd^ antennomeres glabrous, each of the 1^st^ and 2^nd^ with a seta near apex, pubescent from the 4^th^; the 2^nd^ shortest, while 4^th^ longest; relative length of each antennomere compared with the 2^nd^ in the holotype as: the 1^st^ (2.50), 2^nd^ (1.00), 3^rd^ (2.50), 4^th^ (2.93), 5^th^ (2.58), 6^th^ (2.07), 7^th^ (2.29), 8^th^ (2.00), 9^th^ (1.88), 10^th^ (1.64) and 11^th^ (1.71).

***Pronotum*** subcordate, transverse, PL/PW = 0.91–0.92, but a little longer than wide measured through fore angles; much wider but slightly shorter than head, PW/HW = 1.09–1.12, PL/HLl = 0.86–0.96; widest at about 2/5 from front, lateral margins including front and hind angles widely and strongly reflexed throughout, gently and gradually narrowed towards hind angles which are nearly rectangular, fore angle extraordinarily and forwardly protruded, forming a obtuse lobe; basal foveae short but well-marked; only basal latero-marginal setae present, inserted just on the hind angles; entire lateral margins and front without borders, base finely bordered, slightly narrower than front including front angles, PbW/PfW = 0.87–0.88; both base and front nearly straight. Scutellum small.

***Elytra*** elongate, amygdaloid, much longer than wide, EL/EW = 1.80–1.89; distinctly longer than forebody including mandibles, much wider than pronotum; base well-bordered (but unbordered against the 1^st^ interval), shoulders nearly rounded; widest at about middle of elytra, apex distinctly protruded, mucronate; disc convex, marginal depressions well-defined and reflexed throughout, the 9^th^ interval suddenly deepened and distinctly curved at about basal 1/4; striae entire, impressed by small but deep punctures, intervals almost flat; scutellar striole short; basal pores present; the 3^rd^ interval with two setiferous pores close to 2^nd^ stria at about apical 1/5 and 3/7 of elytra respectively; three pores present on 7^th^ stria posteriorly; an apical pore present at apical anastomosis of the 1^st^ and 4^th^ striae; presence of 23–24 marginal umbilicate pores throughout, continuous (Fig. 13A).

***Legs*** slender and elongate, procoxae asetose, mesocoxae unisetose, metacoxae trisetose (the inner setae present); each trochanter with a single seta; metafemur unisetose posteriorly; tibiae and tarsi smooth, without longitudinal sulci or striae externally; the 4^th^ tarsomere bilobed in fore and middle legs, deeply emarginated in hind ones; protarsi not modified in male, but 1^st^ –3^rd^ each with two spongy setae on ventral surface.

Ventral surface smooth and glabrous. Each abdominal ventrite IV–VI bisetose, ventrite VII bisetose in male, quadrisetose in female.

**Male genitalia** (Fig. 14A–D). Median lobe of aedeagus very slender and elongate, gently arcuate in middle portion, then gradually narrowed towards apex which is bluntly pointed; base moderately opened, presence of a small sagittal aileron; parameres developed. In lateral view, apical lobe thin, slightly longer than wide.

##### Remarks.

Similar to *Jujiroa zhouchaoi* Tian & He, 2020 and *J. satoi* Uéno, 2005, but having much longer antennae and distinctly mucronated apices on the elytra. In addition, it is easily distinguished from *J. zhouchaoi* by a broader head, flat intervals and large punctate striae of the elytra and from *J. satoi* by a slenderer body with a thin head, and the presence of dorsal pores on the elytra. Furthermore, *Jujiroa uenoi* sp. nov. has a peculiar character state: presence of an inner seta on each metacoxa, which is absent in other species of *Jujiroa*.

##### Etymology.

Dedicated to the late Dr Shun-Ichi Uéno, Science Museum (Natural History), Tokyo.

##### Distribution.

China (Sichuan). Known only from the cave Banche Dong in Leshan (Fig. 2).

Cave Banche Dong is about 1.6 km away in a straight line from Xiaodouyan Tiankeng (硝斗岩天坑), which is a well-known touristic site in Leshan. The opening of the entrance is so narrow that it allows only one person to crawl in at a time (Fig. 15A). The interior of the cave is small and moist (Fig. 15B). The two individuals of *Jujiroa uenoi* sp. nov. were found running on the ground inside cave (Fig. 15C–D). Other animals observed in the cave are *Leopoldamys* rats, crickets, moths and *Serriphaedusa* snails (Fig. 15E–H).

#### Jujiroa wangzheni sp. nov.

Taxon classificationAnimaliaColeopteraCarabidae

A3FC3A55-A00E-5963-BD8D-559A4E632916

http://zoobank.org/12F04E26-A163-40D0-A8ED-DE16EEA5F6B4

**Figure 16.**
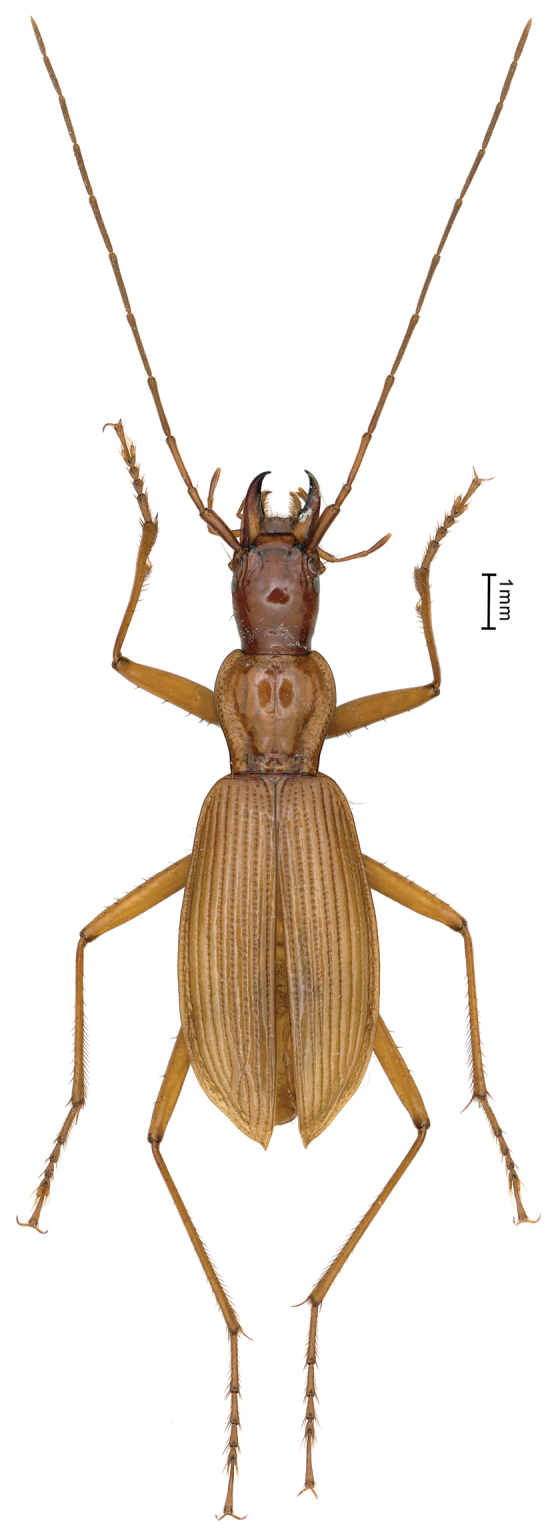
Habitus of *Jujiroa wangzheni* sp. nov., male holotype.

Chinese name: 王震穴胫步甲 Figs 2 , 6 , 13B , 16

##### Material.

***Holotype***: female, cave Hua’er Dong, Xiangdingshan, Xiangding, Shiping, Gulin, Luzhou, Sichuan (四川省泸州市古蔺县石屏镇向顶村象顶山华儿洞), 28.028931°N, 106.00716°E, 640 m, 2020-XI-24, leg. Yuan Li & Zhen Wang, in SCAU. ***Paratype***: 1 female, *idem*, in SCAU.

##### Diagnosis.

A small-sized *Jujiroa* species, depigmented, body and appendages elongate, microphthalmic, head thin and slightly expanded medially, fore angles of pronotum moderately protruded, elytral striae largely punctate, mucronate at apices, absence of dorsal pores, tarsi longitudinally sulcate.

##### Description.

***Length***: 12.5 mm; width: 3.6 mm. Habitus as in Fig. 16.

***Body*** concolorous yellow but a little darker on head, surface smooth and glabrous (though rough on sides and lateral margins of pronotum), moderately shiny. Microsculptural meshes transversely and densely striate on head, pronotum and elytra.

***Head*** thin and elongate, much longer than wide, HLm/HW = 2.00, HLl/HW = 1.48; widest exactly at the eyes location; frons nearly flat, vertex strongly convex medially, neck constriction wide; genae convex, and slightly expanded at side; frontal furrows short and shallow, ending before the level of anterior supraorbital pores; two pairs of supraorbital pores present, anterior near inner margin of eye; eyes very small and flat; clypeus bisetose, labrum bisinuate at front margin, 6-setose; mandibles elongate, teeth reduced; labial suture clear; mentum with two setae on each side, basal pits small; median tooth short, half as long as the lateral lobes, bifid at tip; submentum with two setae on each side, inner ones much longer; ligula short, widened and truncated at apical margin, bisetose; palpomeres long and slender, the 2^nd^ labial palpomere bisetose on inner margin, 1.2 times as long as 3^rd^, the 3^rd^ maxillary palpomere slightly longer than 4^th^; antennae filiform, thin and very long, extended to apices of elytra, the 1^st^ to 3^rd^ antennomeres glabrous, the 1^st^ and 2^nd^ with a seta near apex, pubescent from the 4^th^; the 2^nd^ shortest, while 5^th^ longest; relative length of each antennomere compared with 2^nd^ antennomere in the holotype as follows: the 1^st^ (1.88), 2^nd^ (1.00), 3^rd^ (2.07), 4^th^ (2.89), 5^th^ (2.76), 6^th^ (2.89), 7^th^ (2.35), 8^th^ (2.14), 9^th^ (2.00), 10^th^ (1.57) and 11^th^ (1.64).

***Pronotum*** subcordate, nearly as long as wide, PL/PW = 0.98; wider but slightly shorter than head, PW/HW = 1.38, PL/HLl = 0.88; widest at about 2/5 from front, lateral margins including front and hind angles widely reflexed throughout, gently and gradually straight towards hind angles, fore angles roundly and moderately protruded forwardly, hind angles rectangular; basal foveae large and shallow; only basal latero-marginal setae present, inserted on the hind angles; entire lateral margins and front without borders, base finely bordered, slightly wider than front, PbW/PfW = 1.10; both base and front nearly straight. Scutellum small, short.

***Elytra*** elongate, amygdaloid, much longer than wide, EL/EW = 2.01; distinctly longer than forebody including mandibles, much wider than pronotum; base well-bordered (but interrupted against the 1^st^ interval), shoulders nearly rounded; widest at about 4/7 of elytra from base, apex sharply protruded, distinctly mucronate; disc convex, striae entire, impressed by large and nearly rounded punctures; scutellar striole short; basal pores present; without setiferous pore on the 3^rd^ interval, and absence of the preapical pore; two pores present on 7^th^ stria posteriorly; an apical pore present on each elytron; marginal umbilicate pores present throughout, continuous (Fig. 13B).

***Legs*** very thin and slender, procoxae asetose, mesocoxae unisetose, metacoxae bisetose (without inner setae); each trochanter with a single seta; metafemur bisetose posteriorly; tibiae and tarsi smooth, without longitudinal sulci externally; the 4^th^ tarsomere bilobed in fore and middle legs, while deeply emarginated in hind ones.

***Ventral*** surface smooth and glabrous. Each abdominal ventrite IV–VI bisetose, ventrite VII, quadrisetose.

**Male.** Unknown.

##### Remarks.

More or less similar to *J. deliciola* Uéno & Kishimoto, 2001 (from two caves in Xingwen County, Yibin, southern Sichuan) (Uéno and Kishimoto 2001; Tian and He 2020) in having a thin and elongate body, and distinctly mucronate elytral apices, but *J. wangzheni* sp. nov. is easily recognized by a less shiny and less glabrous body, widened elytra base, strongly convex intervals and largely punctate striae of elytra which are devoid of dorsal pores on 3^rd^ elytral interval and without preapical pores.

##### Etymology.

The new species is dedicated to Mr. Zhen Wang (Chengdu, Sichuan), a co-collector of the type exemplars.

##### Distribution.

China (Sichuan). Known from the cave Hua’er Dong in Gulin County, southeastern Sichuan (Fig. 2).

The specimens of *J. wangzheni* sp. nov. were collected by baited traps in a chamber at about 30–50 m inside of the left entrance of Hua’er Dong. The species is sympatric with *Uenoaphaenops fani* (Uéno, 2003) (Fig. 6E).

### Key to species of the genus *Jujiroa* Uéno, 1952 from Sichuan Province

**Table:** 

1	Large-sized, over 19.0 mm long; interval 3 with five discal pores	***J. lingguanensis* Deuve & Pütz**
–	Medium, or small-sized, less than 16.0 mm long; interval 3 with at most three discal pores	**2**
2	Apices of elytra not mucronate	**3**
–	Apices of elytra distinctly and sharply protruded, mucronate	**4**
3	Head slightly expanded laterally, elytra without dorsal setiferous pores	***J. satoi* Uéno**
–	Head thin, nearly parallel-sided; elytra with two dorsal setiferous pores	***J. zhouchaoi* Tian & He**
4	Eyes slightly convex, pronotum longer than wide	***J. deliciola* Uéno & Kishimoto**
–	Eyes atrophied, flat, pronotum transverse	5
5	Antennae long, reaching the apices of elytra, elytra without dorsal pores along the 3^rd^ stria	***J. wangzheni* sp. nov.**
–	Antennae short, not extending to apices of elytra, elytra with dorsal pores along the 3^rd^ stria	6
6	Metacoxae trisetose (inner setae present), head thin, pronotum with fore angles strongly protruded forwardly, lobed, base narrower than front	***J. uenoi* sp. nov.**
–	Metacoxae bisetose (inner setae absent), head broad, pronotum with fore angles moderately protruded forwardly, not lobed, base as wide as front	***J. iolandae* Vigna Taglianti**

## Supplementary Material

XML Treatment for Uenoaphaenops

XML Treatment for Uenoaphaenops fani

XML Treatment for Chu

XML Treatment for Chu pheggomisetoides

XML Treatment for Boreaphaenops liyuani

XML Treatment for Agonotrechus sinotroglophilus

XML Treatment for Jujiroa uenoi

XML Treatment for Jujiroa wangzheni
